# Informed shared decision-making supported by decision coaches for women with ductal carcinoma in situ: study protocol for a cluster randomized controlled trial

**DOI:** 10.1186/s13063-015-0991-8

**Published:** 2015-10-12

**Authors:** Birte Berger-Höger, Katrin Liethmann, Ingrid Mühlhauser, Burkhard Haastert, Anke Steckelberg

**Affiliations:** University of Hamburg, MIN-Faculty, Unit of Health Sciences and Education, Martin-Luther-King-Platz 6, D-20146 Hamburg, Germany; mediStatistica Neuenrade, Lambertusweg 1b, D-58809 Neuenrade, Germany

**Keywords:** Decision-making, Patient participation, Oncology, Breast neoplasms, Intraductal carcinoma, Evidence-based medicine, Informed choice

## Abstract

**Background:**

Women with breast cancer want to participate in treatment decision-making. Guidelines have confirmed the right of informed shared decision-making. However, previous research has shown that the implementation of informed shared decision-making is suboptimal for reasons of limited resources of physicians, power imbalances between patients and physicians and missing evidence-based patient information. We developed an informed shared decision-making program for women with primary ductal carcinoma in situ (DCIS). The program provides decision coaching for women by specialized nurses and aims at supporting involvement in decision-making and informed choices.

In this trial, the informed shared decision-making program will be evaluated in breast care centers.

**Methods/Design:**

A cluster randomized controlled trial will be conducted to compare the informed shared decision-making program with standard care. The program comprises an evidence-based patient decision aid and training of physicians (2 hours) and specialized breast care and oncology nurses (4 days) in informed shared decision-making. Sixteen certified breast care centers will be included, with 192 women with primary DCIS being recruited.

Primary outcome is the extent of patients’ involvement in shared decision-making as assessed by the MAPPIN-O_dyad_ (Multifocal approach to the ‘sharing’ in shared decision-making: observer instrument dyad). Secondary endpoints include the sub-measures of the MAPPIN-inventory (MAPPIN-O_nurse_, MAPPIN-O_physician_, MAPPIN-O_patient_, MAPPIN-Q_nurse_, MAPPIN-Q_patient_ and MAPPIN-Q_physician_), informed choice, decisional conflict and the duration of encounters.

It is expected that decision coaching and the provision of evidence-based patient decision aids will increase patients’ involvement in decision-making with informed choices and reduce decisional conflicts and duration of physician encounters. Furthermore, an accompanying process evaluation will be conducted.

**Discussion:**

To our knowledge, this is the first study investigating the implementation of decision coaches in German breast care centers.

**Trial registration:**

Current Controlled Trials ISRCTN46305518, date of registration: 5 June 2015.

**Electronic supplementary material:**

The online version of this article (doi:10.1186/s13063-015-0991-8) contains supplementary material, which is available to authorized users.

## Background

Women with breast cancer want to participate in treatment decision-making [1–3]. The German guideline recommends mastectomy or breast-conserving surgery combined with (neo-) adjuvant treatments [4]. Benefits and harms of treatments vary between options and differently impact patients’ physical and psychosocial quality of life [5–7]. Therefore, women’s values and preferences are essential. Treatment decisions should not be left exclusively to the physicians [8].

So far, women’s preferences to participate have rarely been considered in a structured manner [3]. This might contribute to dissatisfaction with medical treatment decisions [9]. The National Cancer Plan of the German Ministry of Health and breast cancer treatment guidelines explicitly ask for patient participation in medical decision-making [4, 6, 10, 11]. In addition, the German patients’ right act clearly defines patients’ rights to complete and unbiased information on benefits and harms of all treatment options including the option not to treat [12].

Evidence-based patient decision aids (ptDA) and the consideration of women’s preferences are key issues in decision-making [13]. Some women feel overloaded by the plethora of information, usually given in a single consultation [14]. The model of shared decision-making (SDM) comprises evidence-based patient information and weighing of pros and cons of treatment options while taking women’s values and preferences into account. Barriers that might hamper the implementation of informed SDM have been identified. They comprise structural deficiencies in care settings, communication or time issues [15, 16]. Patients experience discontinuity in the process of care [14, 15] possibly due to a substantial mismatch in knowledge, different role models or social and cultural backgrounds [2, 15]. Patients often do not feel prepared to participate in decision-making due to their limited knowledge. In SDM, acknowledgement of patients’ preferences and values is as important as knowledge about treatment options [17]. Even if women decline to participate in decision-making, they often wish detailed information about their disease and treatment options [18, 19]. In case of declining, the decision can be delegated to the health care team at any time. SDM comprises the sharing of the decision-making process, but not necessarily the sharing of the decision-making itself [20]. Moreover, women have the right of non-information.

### State of research

#### Shared decision-making and decision coaching by specialized breast care nurses

One opportunity to overcome some of these barriers is the inter-professional model of informed shared decision-making that facilitates patients’ involvement in treatment decision-making [21].

Health care professionals support the decision-making process as decision coaches, which may be defined as “*health professionals who provided information on options and facilitated progress in decision making in preparation for discussion with the practitioner who would ultimately be responsible for making the decision with the patient”* [22].

The majority of projects on SDM have focused on the education of the physicians [23–25]. Some studies have shown that specialized nurses who acted as decision coaches successfully enhanced patients’ decision-making [22, 26–28]. Nurses have shown competences in explaining medical information, supporting patients and sharing the information with physicians [15]. At the Dartmouth-Hitchcock-Medical-Center in New Hampshire, USA, specially trained nurses and social workers prepare patients for decision-making in a structured manner. A consultation in the hospital’s “Center for Shared Decision Making” is mandatory, for example, for breast cancer patients who take part in the *Comprehensive Breast Program*. This includes a pre-visit distribution of decision aids into the standard care path and a continuing care coordinator who guides patients through the decision and care process [29, 30].

In Germany, nurses have already been involved in patient education in a structured manner within the scope of the disease management programs for diabetes mellitus, asthma and hypertension [2]. So far, neither the implementation of SDM [24, 31] nor SDM involving breast care nurses or oncology nurses as decision coaches [22, 27] has been implemented in oncology care settings. Specialized nurses successfully completed at least an additional 1-year training in care of breast cancer (oncology) patients [32–34]. SDM has not yet been included into the curricula [33].

#### Effects of evidence-based patient decision aids on patient participation in decision-making

Evidence-based patient information (EBPI) has frequently been neglected in the scope of SDM. EBPI is a prerequisite for informed SDM processes [35, 36]. The combination of SDM and evidence-based ptDA enables women to make informed choices defined by Marteau as relevant knowledge about the treatment options and a positive attitude congruent to their chosen treatment [27, 37–39]. Previous research has shown that the exclusive provision of written ptDA improves breast cancer patient’s knowledge and reduces decisional conflicts [40, 41]. However, the exclusive provision of evidence-based ptDA for patients is not enough to enhance patient empowerment [42]. In Germany, strategies to improve evidence-based patient participation have been explored, for example, within the care of multiple sclerosis patients. Kasper et al. [42] evaluated an evidence-based ptDA on immunotherapy in patients with multiple sclerosis. The study could not show a difference in preference matches between intervention and control group. Köpke et al. showed that an interactive educational intervention increased informed choices and slightly increased the autonomy preference toward an active role in patients [43]. According to the Theory of Planned Behavior (TPB) autonomy preference only indicates behavioral intention and not the behavior itself [44]. The TPB explains how peoples’ behavioral intentions are influenced by social norms, their attitudes toward the behavior and their perceived behavioral controls. The behavioural intention is strongly associated with the prediction of the behavior itself [44].

Collins et al. [41] reported that a decision aid on breast cancer reduces decisional conflict in women facing a treatment decision on surgical options.

Within the scope of the German healthcare system, evidence-based treatment and patient guidelines for breast cancer have been developed [4]. However, these guidelines do not provide adequate risk communication on treatment options and especially harms. PtDA that adequately consider criteria for EBPI are still rare within the German healthcare system [45].

#### Evaluation of SDM

Measurements for SDM in inter-professional settings remain challenging. A variety of instruments exist to measure preconditions, the SDM-process and outcome parameters [46]. However, none of these instruments focus on the inter-professional specifics of measuring team processes and triangular SDM-processes among, for example, doctors, nurses and patient.

### Previous work

#### Development

We developed a program (decision coaching) to facilitate informed SDM in oncology. The decision coaching will be provided by specialized breast care or oncology nurses combined with evidence-based ptDA. We address women who are facing a primary treatment decision on ductal carcinoma in situ (DCIS).

We chose DCIS, which is associated with a great extent of uncertainty. Women with DCIS have distinct information needs about the disease and its treatment options [47]. Every year 6,500 women are diagnosed with DCIS in Germany. The number of cases has been increasing since the introduction of the national mammography screening program [48]. Without screening, DCIS is rarely diagnosed. Due to its symptomless nature, it usually is an incidental finding. Approximately, 20 % of the malignancies detected by screening are DCIS [49, 50]. DCIS is limited to the mammary ducts and associated with an increased risk of invasive breast cancer. The prognosis is unknown, but not all DCIS will progress to invasive breast cancer [51]. Treatment is usually recommended for all woman with DCIS because it is impossible to predict which DCIS will develop into invasive breast cancer [4].

We developed our program following the UK Medical Research Council’s (MRC) guidance for the development and evaluation of complex interventions [52] and applied the Theory of Planned Behavior. Our program comprises an evidence-based ptDA [53], a curriculum for specialized nurses and a workshop for physicians [53, 54]. We developed our curricula according to the six-step approach for the development of curricula in medical education [55]. The evidence-based ptDA for the primary treatment decision on DCIS has been developed according to the criteria for EBPI [38].

#### Pilot trial

Each component of the intervention was separately piloted [54]. Finally, feasibility and acceptability of the complete program has been evaluated in a phase II study [53].

Two breast care centers (four nurses and five physicians) in Berlin, Germany, recruited up to 6 patients with DCIS [53]. All encounters with patients and nurses were videotaped and analyzed. In sum, the study showed that the program is feasible and acceptable. However, we also identified barriers. The physician workshop revealed the physicians’ desire to discuss treatment options; in standard care, usually only the recommendation of the tumor board is communicated to the patient, which is in line with medical guidelines. This option is often seen as the only possible treatment. Therefore, an open decision-making process is inhibited. Physicians also unveiled patients’ supposed expectation of doctors. According to their opinions, patients expect clear treatment recommendations. Both professional groups - nurses and physicians - pointed out that it is not necessary to ask patients for their preferences because they already know which option would be the best for the patients. Although each woman fulfilling the inclusion criteria should have been asked for study participation, not all women were asked for participation because physicians were concerned that some women might be overburdened by SDM due to age or educational background. Another barrier consists in the predefined number of specific surgical procedures that lead to a doubtful incentive for breast care centers. Physicians were concerned about offering women all treatment options since they had to justify their treatment to the certification body that defines the quality indicators.

We address the potential barriers in our educational intervention by discussing the possible impact of personal attitudes and giving advice on how to communicate the recommendation of the tumor board. In addition, we developed supplementary material such as prompt cards and fact sheets to structure the decision coaching sessions.

### Objectives

This study aims to assess efficacy of the decision coaching program. The key hypothesis is that women who receive an evidence-based ptDA and decision coaching by specially trained breast care or oncology nurses are more involved in decision-making compared to standard care.

We also hypothesize that women in the intervention group will make more informed choices based on their preferences and values.

Further objectives are to assess if women’s decisional conflicts will decrease and if the duration of physician encounters will be shortened.

Alongside the trial, the processes, facilitators and barriers to implementation will be qualitatively assessed.

## Methods/Design

This protocol is reported following the criteria of the standard protocol items: recommendations for interventional trials (SPIRIT) [56] and the MRC-Framework for the development and evaluation of complex interventions [52]. For the completed SPIRIT checklist see Additional file 1.

### Trial design

The intervention will affect the structures and procedures of the breast care centers. To avoid contamination, allocation will be carried out on the level of the breast care centers rather than the individual patient [57]. The trial is designed as a superiority cluster randomized controlled trial with a parallel group design and 1:1 allocation ratio.

### Study setting

The study will be conducted in certified breast care centers. Breast care centers in Germany in the federal states of Schleswig-Holstein, Hamburg, Bremen, North Rhine-Westphalia, Hessen, Saxony-Anhalt, Mecklenburg-West Pomerania and Lower Saxony will be invited for participation by sending an email with brief information and personal contact details. Usually, breast cancer patients are referred to breast care centers to verify diagnosis and provide treatment. Certified centers offer quality assured care based on evidence-based medical treatment guidelines [10, 11]. That implies that breast care centers have to treat a predefined number of patients per year. The diagnoses and treatment recommendations have to be made by a multi-professional healthcare team. Usually, the tumor board’s treatment recommendation is made prior to the primary treatment of DCIS and before talking with patients about treatment options. In most breast care centers, patients are only offered this treatment recommendation. Breast care centers are continuously evaluated, using predefined quality indicators [58, 59]. Certification guidelines require the involvement of breast care nurses or oncology nurses in patient care. In sum, certification allows comparability of the participating centers.

### Eligibility criteria

Breast care centers are eligible if they have been certified according to a German or European certification body (such as Onkozert, Äkzert or EUSOMA). Breast care centers have to agree to release the specialized nurses from duty to allow participation in physician consultations and tumor boards and to conduct decision coaching sessions. Physicians have to be dispensed for the workshop.

Nurses are eligible if they have a 1- or 2-year advanced training as breast care nurses or oncology nurses or if they have been entrusted with breast care nurses’ tasks for at least 6 months.

Physicians are eligible if they are involved in the information process of women facing a primary treatment decision on DCIS.

Women aged 18 years or older with a primary, histologically confirmed DCIS in the absence of invasive breast or lobular carcinoma in situ and facing a primary treatment decision will fulfil the inclusion criteria. Since all the information is provided in the German language, patients need sufficient language skills.

Written informed consent will be obtained from physicians, nurses and women.

### Patient exclusion criteria

Women who are pregnant, have a known BRCA 1/2 mutation or who had a previous diagnosis of breast cancer or DCIS (irrespective if ipsi- or contralateral) are not eligible. Furthermore, women with contraindications for radiotherapy will not be included. Women seeking a second opinion will be excluded to avoid contamination of the study arms since participating women are allowed to consult a further breast care center for a second opinion.

### Interventions

#### Intervention clusters

##### Nurse training

The nurse training lasts 4 days and comprises two modules. The modules contain basics of evidence-based medicine and EBPI including risk communication, an introduction into the developed evidence-based ptDA for women with DCIS and a structured training in SDM [53]. In addition to lectures and presentations, role plays are used to acquire decision coaching skills. Prompt cards, a patient decision guidance and fact sheets will be used to structure decision coaching sessions and ensure that coaching is delivered as intended. Decision coaches will receive a folder comprising the educational material with additional literature. The nurse training will be delivered near the nurses’ workplaces. The group size will not exceed eight participants. The training success will be surveyed using exercises and knowledge tests, which will be analyzed and discussed with participants. The decision coaches receive a structured feedback for the first two decision coaching sessions with women in their centers.

##### Physician workshop

The workshop for physicians lasts 2 hours. It provides an overview of the SDM concept and delivers basics concerning relative and absolute risk reduction. The possible treatment options and its evidences are discussed. Furthermore, participants receive a workshop manual, the evidence-based ptDA and gain insight into the decision coaching sessions. In sum, the physicians get the information that their patients will be given before their consultation and are instructed on their role within this process. The workshop will be delivered in each breast care center randomized to the intervention group (IG). The number of workshop participants is not restricted.

All trainings and workshops will be conducted by researchers of the University of Hamburg (BBH, KL and ASt) MIN-Faculty, Unit of Health Sciences and Education.

##### Evidence based ptDA

The ptDA contains evidence-based information on DCIS, the natural course of the disease, and treatment options including benefits and harms. Due to the paucity of randomized controlled trials, the uncertainties about harms and benefits of treatment options remain high. Existing randomized controlled trials insufficiently report the harms of treatments [53]. We also included the option “watchful waiting” in our ptDA although we have no credible data and predictors to estimate how often and under which conditions an invasive breast cancer will develop. We therefore communicate the risks that come along with this procedure in detail. Data on frequencies will not be available until the results of the ongoing LORIS-trial will be published. This RCT has recently started and compares active monitoring with surgery. It will provide at least some answers [60].

##### Patients

**Fig. 1:**
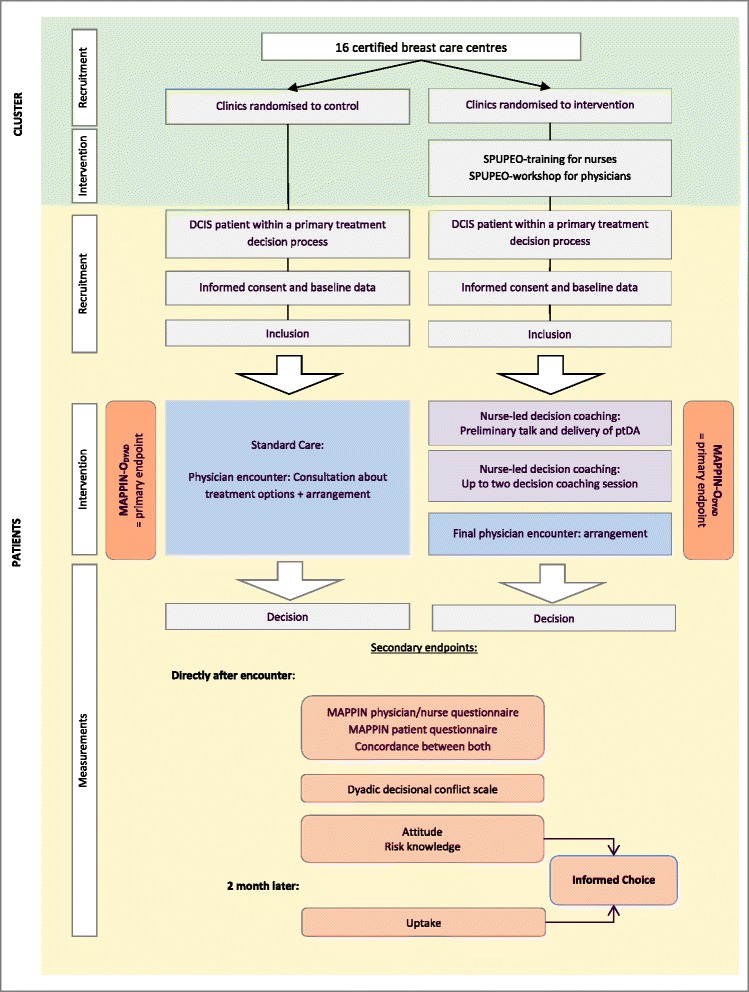
Participant timeline

Women are offered a decision-making process supported by an **evidence-based ptDA**, at least one **decision coaching session** with a trained nurse and a final physician encounter (see Fig. 1).

Nurses will provide information on the **decision coaching,** the **evidence-based ptDA** and the decision guidance that is targeted to the decision to be made. They will also arrange a new appointment within 1 week for the decision coaching and provide her contact details. At the next appointment, the nurse supports the woman’s decision-making process in a structured manner, considering the six steps of SDM [61, 62]:

1. Definition of the problem requiring a decision-making process
2. Key message (There is more than one option, and the best option depends on how the patients value the evidence, the benefits and harms considering their expectations and preferences.)
3. Information about the options, including benefits and harms based on EBPI
4. Clarifying patient’s values and preferences
5. Decision-making (optional to postpone the decision)
6. Arrangement

Nurses will carry out the first five steps. If the woman is not ready to make a decision yet, an additional decision coaching session will be offered. If the woman makes her decision, the nurse will arrange the physician consultation in which physician and woman discuss the preferred option and talk about remaining questions. Patients and physicians will make an arrangement about treatment or watchful waiting (sixth SDM-step) and possibilities to evaluate the decision. Women are allowed to revise their decision at any time if they are not satisfied with their initial decision.

#### Control cluster

In the control cluster, standard care will be delivered. Women will not receive any additional information or counselling on treatment options other than that usually provided by breast care centers. In general, standard care comprises one or two physician encounters in which the women are informed about their diagnosis and the treatment recommendation of the tumor board and their informed consent for treatment is obtained. Standard care procedures of each breast care center will be assessed at the beginning of the study. We expect centers to be comparable due to certification guidelines.

### Criteria for discontinuation

#### Adverse events

We do not expect adverse effects caused by decision coaching. However, uncertainty of treatment options for DCIS is high. The natural course of the disease is unknown [51, 63]. Since it is impossible to predict which DCIS develops into invasive breast cancer, medical treatment guidelines recommend that all women with DCIS should be treated. In principle, every woman has the right to decline treatment. In our pilot study, physicians and nurses reported on women who have chosen this option, which often resulted in high social pressure by healthcare professionals and relatives. As a result, women may refuse further surveillance for fear of being rejected by physicians.

Uncertainties related to treatment options might frighten women. On the other hand, the entire information including pros and cons and nurses’ guidance through the decision-making process could decrease uncertainty in women [64].

We do not expect harm. In addition, psycho-oncological support has been implemented into standard care. Women will also be informed about this proposition in the study information sheet. Beforehand, the psycho-oncologist will be informed about the study.

#### Participant withdrawal

All participants will receive written and verbal information about their opportunity to withdraw from the study at any time without any consequences, and in that event, the participant’s data will be deleted. Participant withdrawal is possible under specification of the participant’s code number. A withdrawal of anonymized data is not possible.

### Baseline data

The following data will be surveyed from breast care centers before randomization: Primary cases of DCIS treated with (1) breast-conserving therapy, (2) additional recommendation of radiotherapy afterwards and (3) mastectomy in 2014, certification body (Onkozert, EUSOMA, ÄKzert), number of specialized nurses in the breast care centers, number of treating physicians and procedures in the breast care center.

Baseline characteristics of specialized nurses include age, gender, working experience, training and education, job specification, knowledge about DCIS, experiences in patient counselling, participation in physician consultations and tumor boards and nurses’ understanding of their role.

Baseline characteristics of physicians include age, gender, working experience in senology, qualification, attitude towards the inter-professional collaboration and knowledge about evidence-based medicine and SDM.

Baseline characteristics of women with DCIS include age, educational level, disease and diagnostic parameters (size of DCIS, histologic type = grading, architecture, focality, hormone-/HER2-receptor-status if available, comorbidities, mode of detection) (all diagnostic parameters will be extracted from the patient record), information about health information behavior, and advance information about DCIS.

All baseline characteristics except for the women will be assessed before randomization to avoid bias.

### Outcomes

#### Primary outcome: MAPPIN-O_dyad_

Different outcomes have been discussed. For the evaluation of our main hypothesis, we will use the observer instrument “Multifocal APProach to the sharing IN Shared Decision-making” (MAPPIN’SDM) [23]. As an instrument for the assessment of the primary outcome the observer-based instrument MAPPIN-O_dyad_ of the MAPPIN-inventory will be applied. It assesses the mutual SDM behavior of physicians and patients based on video recordings.

Informed choice would have been a suitable endpoint, too. The intervention addresses the enhancement of patient participation, which is part of SDM. The estimation of patient participation requires an analysis of the decision-making process and its communicative details. Informed choice is an outcome measure, which correlates with patient participation, but it is not congruent. Therefore, the process itself is the primary endpoint of the study, and further outcome variables have been defined as secondary endpoints. A list of all outcomes is provided in Table 1.

**Table 1:**
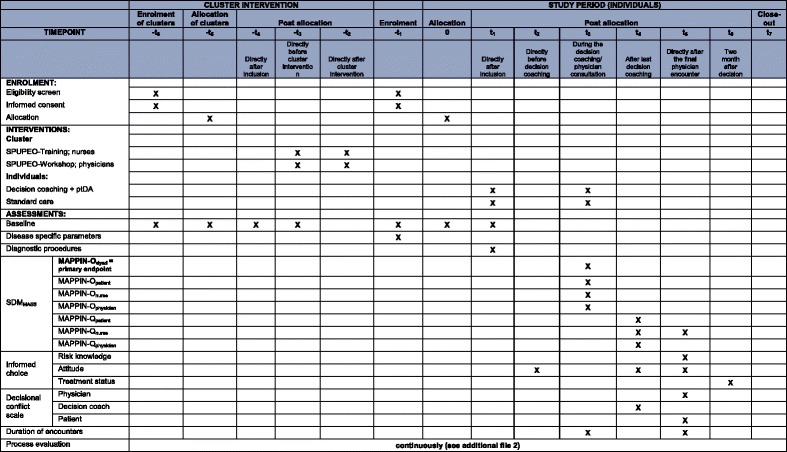
Outcome parameters

MAPPIN-O_dyad_ is one part of the MAPPIN’SDM inventory that enables the appraisal of medical decision communication with three perspectives: physician, patient and observer. The aim of the instrument is to assess the extent of patient participation. All three perspectives consist of the same SDM criteria contained in analogous items. The relevant evaluation unit is the dyad of physician and patient. Physician and patient assess how they perceived the mutual SDM process. The observer rates the SDM behavior of the physician and patient and of the interaction of the dyad (physician and patient) based on video recordings.

The multifocal approach with dyad as evaluation unit is unique for SDM process assessment inventories. Other instruments either skip at least one perspective (for example, SDM-Q) [65, 66] or focus the action of only one conversational partner, mostly the physician (for example, dyadic OPTION, OPTION 5) [67–69]. In fact, a triangular measurement of SDM including the whole decision-making process and all participants (physician, nurse and patient) would be desirable. However, such instruments do not exist [46]. Originally, MAPPIN’SDM has been developed to assess the communication between physician and patient. Meanwhile an adapted version for the decision talk between health care professionals and patients has been published [70]. Therefore the observer-based MAPPIN’SDM-inventory (MAPPIN-O_dyad_) considering the interaction of the patient with both, the physician and the nurse, will give a holistic view of the SDM process (see below). The decision-making process in the IG comprises the decision coaching by the nurse and the patient-physician consultations. In the control group (CG), the decision-making process comprises the patient-physician consultations.

The revised version of the instrument (MAPPIN’SDM_revised_) is available, comprising a set of nine SDM indicators per perspective, which cover the international consensus of the essential SDM competencies [71].

Both MAPPIN’SDM questionnaires (healthcare provider and patient) and the observer instrument comprise 11 items (one indicator has three components). Six indicators outline the chronological order of an SDM talk. Three indicators contain meta-communicative components. All indicators are assessed on a five-point-scale from 0 to 4 (for example, observer instrument: ‘competence was not observed’ to ‘excellent performance’).

For the primary outcome in the IG, two trained raters will independently rate MAPPIN’SDM_dyad_ for the whole decision-making process, consisting of two talks (nurse-patient and physician-patient). In a second step, they build a consensus. In the last step, we will calculate mean values of the final scores for the primary endpoint.

In the CG, the decision-making process is limited to the patient-physician consultations. The rating procedure is identical to the IG, but the decision-making process comprises only one consultation (physician-patient).

Test quality of an observer-instrument consists of several data. The typical item parameters as well as the reliability of the questionnaire are adequate (mean item difficulty = 1.51 (SD = 0.76), mean corrected item total correlation = 0.53 (SD = 0.19), Cronbachs alpha = 0.84). The reliability and validity of the observer data are relevant as well. Previous research has shown adequate interrater-reliability (mean T = 0.45). Validity calculations were conducted by sensitivity and specificity measurement in comparison to one SDM-expert. High validity values were reached (mean sensitivity = 0.67, mean specificity = 0.81) [72].

#### Secondary outcomes

##### MAPPIN-Q_(patient/nurse/physician)_

To take patients’ and health care professionals’ perspective of SDM into account, the remaining components of the MAPPIN’SDM-inventory will be used. This comprises women-, nurse- and physician-based judgments about their perceptions of the mutual decision-making process (MAPPIN-Q_patient_, MAPPIN-Q_nurse_ and MAPPIN-Q_physician_, respectively). The healthcare professionals will be familiar with the questionnaire because they fill in the questionnaire for every woman. Patients in the IG fill in the questionnaire two times (talk with nurse, talk with physician), and patients in the CG fill in the questionnaire once.

The concordance between SDM judgment of patient and physician/nurse by a nonparametric concordance measure will be calculated (weighted T (MAPPIN-Q_patient_ and MAPPIN-Q_physician_) and weighted T (MAPPIN-Q_patient_ and MAPPIN-Q_nurse_)).

MAPPIN-O_dyad_ of the decision coaching sessions and MAPPIN-O_dyad_ of the physician encounter will be reported separately, as well as the observer ratings of patients and healthcare professionals (MAPPIN-O_patient_, MAPPIN-O_nurse_ and MAPPIN-O_physician_).

Every part of the MAPPIN’SDM-inventory has 11 items and answer format of a graduated Likert scale from 0 to 4 (see primary outcome). The test values per person are calculated by the mean of the 11 item answers. All MAPPIN’SDM-ratings and -questionnaires that are applied for secondary outcomes have similar test quality as the primary outcome [73].

##### Informed choice

Following up Marteau’s definition of informed choice, the measure of this construct combines three dichotomous dimensions: (a) risk knowledge, (b) attitude and (c) uptake [37]. These three dimensions will be connected to one dichotomous outcome. Informed choice can be obtained if women have adequate risk knowledge of the treatment options, and their uptake of one treatment option is congruent with their positive attitude for the chosen option (see Table 2) [37].

**Table 2:** Classifying choices based on the three dimensions of informed choice

Uptake	Good knowledge	Positive attitude toward the option	The choice is
Mastectomy	✓	✓	Informed
	✕	✓	Uninformed
	✓	✕	Uninformed
	✕	✕	Uninformed
Breast-conserving surgery without radiotherapy	✓	✓	Informed
	✕	✓	Uninformed
	✓	✕	Uninformed
	✕	✕	Uninformed
Breast-conserving therapy and radiotherapy	✓	✓	Informed
	✕	✓	Uninformed
	✓	✕	Uninformed
	✕	✕	Uninformed
Watchful waiting	✓	✓	Informed
	✕	✓	Uninformed
	✓	✕	Uninformed
	✕	✕	Uninformed

The single dimensions of the instrument are described below:

- Risk knowledge
  A 12-item knowledge test has been developed to measure risk knowledge. The test includes questions on diagnosis and prognosis of DCIS and benefits and harms of available treatment options in multiple-choice format. It was pretested in the pilot trial. However, it cannot be considered a validated scale. The score is calculated by the sum of correct answers (maximum value 12). Total values will be dichotomized in adequate risk knowledge (at least 75 %; ≥ 8 correct answers) and non-adequate knowledge (<8 correct answers). If patients fill in at least one item of the risk knowledge test, unanswered questions will be rated as incorrect. If none of the questions is answered, the test will be classified as missing data. All patients will fill in the risk knowledge test after their last physician encounter to avoid questions from patients triggered from the test items.
- Attitude
  The attitude of patients toward the options will be assessed during the SDM process. Because the decision can be influenced by several factors, the attitude in the IG will be assessed at baseline, before the first decision coaching session (after reading the decision aid), after the last decision coaching session and after the final physician encounter in which the treatment decision is made. In the CG, attitude will be assessed before (baseline) and after the physician encounter. The questionnaire comprises the attitude for every option using a four-point Likert scale (“non-sensible” to “sensible”). We will only include the score regarding the treatment option that was finally chosen by patients in the measure of informed choice. The further judgments will be assessed in the scope of the process evaluation. The scale is newly developed.
- Uptake
  Uptake indicates whether women stick to their decisions made with the medical team (nurses and physicians) within the decision-making process. This information will be extracted from patient records 2 months after the final physician encounter and is included in the measure of informed choice.

To get a dichotomous value for informed choice, we classify women with adequate knowledge and positive attitude toward the chosen option as making an informed choice. Additionally, all sub-dimensions of informed choice (uptake, risk knowledge and attitude) will be analyzed separately. It is possible that diagnosis changes after the decision-making process, for example, if an invasive cancer is detected post-surgery. This changes the preconditions for the treatment decision as well. In such case, the initially favored treatment option (for example, to dispense with radiotherapy) will not be classified as uninformed; instead, the decision will be rated only if the preconditions for the treatment decision (= DCIS) did not change.

##### Decisional conflict

Decisional conflict of patients will be measured using the decisional conflict scale (DCS). Decisional conflict is a condition in which uncertainty about the right decision exists [74]. The DCS includes five subscales: personal uncertainty, modifiable deficits of feeling uninformed, unclear values, inadequate support and perception that an ineffective choice had been made. Sixteen items are scored on a five-point Likert scale (1 = strongly agree to 5 = strongly disagree).

A patient and physician version is available containing the same items (dyadic DCS) [75]. In this study the dyadic version is used and will be supplemented with a nurse version. A German version of the DCS has been validated with 1,286 patients and yielded good test quality [76]. Due to some imprecise item wordings, an adapted physician version will be used that has recently been developed and validated by Kasper et al. but has not been published yet [77, 78].

#### Further parameters

##### Duration of consultations

We will assess the duration of decision coaching sessions and physician encounters and the interval between the encounters.

In addition, data from all participants will be collected during the accompanying process evaluation.

### Procedures and data collection

#### Identification of eligible patients

Potentially eligible women will be identified during the tumor boards or by upcoming appointments. To avoid selection bias, an independent person will contact participating breast care centers (specialized nurses) weekly after the tumor boards to identify eligible patients. Nurses will be instructed to alert physicians of eligible patients. The number of primary cases within the study period will be compared to the number of patients identified as eligible and asked to participate. Physicians of the participating breast care centers will fill in a recruitment form. If inclusion criteria are met, women will be invited to participate in the study, and their informed consent will be obtained (see Fig. 1). To avoid recruitment bias, physicians receive a guideline to invite women with DCIS in a standardized manner. Reasons to decline study participation will be documented if unveiled.

#### Baseline data and allocation

Women in the IG will fill in the baseline data and attitude toward treatment. Completion of questionnaires will require 5 min. Afterward, women receive the DCIS-ptDA and the patient decision guidance.

For women in the CG, the physician encounter will be interrupted and women are invited to fill in baseline data forms. Women are given enough time to think about study participation. If they decide to participate, the patient-physician encounter will continue according to the study protocol.

#### Encounters one and two

Multiple assessments of the decisional conflict scale and the MAPPIN-Q_patient_ can influence the results. Similar preconditions between professionals and women, as well as between the IG and CG, may avoid this bias. Therefore, women receive these questionnaires for reading before the encounters with nurses and physicians. Before the coaching session (and after reading of the ptDA), the patient’s attitude toward treatment options will be assessed again. The reading and completion of questionnaires will require 5 min. After the last decision coaching session, patients in the IG will complete the MAPPIN-Q_patient_, the DCS and the attitude scale. Completion will require about 10 min.

Patients in the IG will complete the remaining questionnaires, including the risk knowledge test after the final physician encounter, which requires about 15 min.

In the CG, patients will complete MAPPIN-Q_patient_, the attitude towards decision questionnaire, the DCS and the risk knowledge questionnaire after finishing the physician encounter (15 min).

After the final encounter with patients, nurses in the IG and physicians in IG and CG will fill in the MAPPIN-Q_physician/nurse_ and the DCS, respectively (5 min).

Women will complete all questionnaires during their appointments in the breast care centers to ensure completeness. If necessary, women will be provided with a prepaid envelope.

If women do not complete all forms, they will be contacted by the study nurse via telephone. Women will be asked to fill in the forms at their next appointment. If the decision-making process was already completed, missing forms will be send to the woman by post.

We do not expect women to miss an appointment because often women feel time pressure for decision-making due to the threatening diagnosis. Women would be contacted by decision coaches to arrange a new appointment if they missed one.

All encounters will be video recorded by nurses and physicians. The video camera will not focus on patients but on the desk with the coaching material. The recordings will be rated independently with the MAPPIN-O_dyad_by two raters. Discrepancies will be solved by discussion after finishing the rating respectively.

The final choice of women will be assessed 2 months after the treatment decision was made.

### Sample size

The sample size calculation is adapted to the primary outcome (MAPPIN-O_dyad_) and is conducted under the assumption of the same variances in both groups for a continuous normally distributed target variable of a two-arm cluster randomized controlled trial.

The assumption on the expected difference between IG and KG and the possible size of the cluster effect is based on previous research [77, 79–81]. A difference of 0.25 in the primary outcome should be revealed between the groups with a power of 90 % and an assumed standard deviation of 0.4. Because we could not identify reliable estimates of the intracluster correlation coefficient (ICCC), we estimate an intracluster correlation coefficient of 0.02 based on previous studies in stroke units and hospitals and due to the fact that breast care centers feature a high standardized care process through the certification [79, 80]. The cluster size will be m = 12 (12 patients per breast care center). As a primary test, a cluster-adjusted t-test according to Donner et al. [82] will be used. The calculation results in a sample size of 14 breast care centers with 168 patients based on an alpha of 0.05. Under consideration of drop-outs, 16 breast care centers will be recruited with a total sample size of n = 192 patients.

### Recruitment

Breast care centers that have at least 20 primary cases of DCIS per year will be invited for study participation. Breast care centers and potential participating health care professionals will be informed about the study. Each breast care center will recruit 12 patients within 9 months.

The recruitment of women will be conducted by the physicians of the breast care centers.

### Allocation

The statistician (BH) has provided a computer-generated allocation sequence. A random permuted block design using blocks of sizes 4, 6 or 8 will be used. No stratification is performed. An independent external person will unveil the allocation after inclusion of all breast care centers. Corresponding to the cluster design, breast care centers will be aware of their allocation status. To avoid selection bias, patients will not receive information about their allocation status. Women will be asked to assume which kind of counselling procedure they received because the unveiling of allocation could have influenced their responses.

If patients would have information about their allocation status before participation, they might not agree to participate or a nocebo-effect might occur in the CG due to the assumption that their treatment might be inferior to the IG. As opposed to this, patients in the intervention group might not agree since they estimate for example, the expenditure of time too high or they would insist on immediate treatment decisions by physicians.

### Blinding

Clusters and patients cannot be blinded (see above). Due to the obviousness of the material used in the IG (for example, prompt cards, decision guidance and fact sheets), video raters cannot be blinded. However, the members of the research team who enter the data are blinded to allocation. The analyst will conduct a blinded review of data before the study groups are unveiled, and the final statistical analysis will be conducted.

### Data management

Data will be administered and entered by the team members of the Unit of Health Sciences and Education of the University of Hamburg. Each study participant will receive a pseudonym to allow for combining of data sets of different time points. Data entry will be verified by two blinded members of the research team. Data will be stored for 10 years as suggested by the German Research Foundation (DFG) [83].

### Statistical methods

#### Statistical methods for analysing primary and secondary outcomes

Data analysis will be conducted following the intention-to-treat principle.

The patient participation (MAPPIN’SDM) in the decision process will be estimated by the primary outcome. Mean values and cluster-adjusted standard deviation [82] in IG and CG will be estimated and compared with a cluster-adjusted t-test. Cluster adjustment is performed using the intracluster correlation coefficient as described in detail by Donner et al. [82]. Linear mixed models including clusters as random effect would be an alternative model [82]. Using a simple design, as in this trial, similar results would be expected for both methods [82]. We decided to use the simpler method of a cluster-adjusted t-test, which has been used in many other cluster randomized controlled trials before.

For all further MAPPIN-questionnaires (secondary outcome) and ratings (inclusively concordance), cluster-adjusted t-tests are also used under the same assumption as described before.

Sub-analysis will be conducted for physician and nurse encounters separately for example, if some of the indicators of MAPPIN could be observed more frequently in nurse or in physician encounters.

Likelihoods of informed choices will be estimated by cluster-adjusted confidence intervals and compared by a cluster-adjusted chi-square-test [82].

Additionally, as secondary analysis, the risk knowledge will be compared nonparametrically between the IG and CG by a Wilcoxon-test on cluster-level (as a non-dichotomized measure).

As secondary analysis decisional conflict (DCS) will be compared by a cluster-adjusted t-test.

All tests will be carried out two-tailed with a significance level of α = 0.05, confidence intervals will be calculated as two-tailed 95 % confidence intervals.

#### Methods for any additional analyses

Baseline data of both groups will be analyzed descriptively. To ensure test quality, the used instruments will be checked by item analysis that is oriented on the common parameters in literature [84].

Furthermore, it will be assessed descriptively, whether women in the IG choose less invasive treatments more often compared to women in the CG.

#### Handling of missing data

Only a few missing values of indicators are expected for the primary outcome because the video rating by the trained observers is very dependable. Missing values are only expected if physicians or nurses start the video too late or stop it too early, thereby missing important information discussed with patients. In those cases of missing values, the total score will be calculated as a mean of the remaining ratings of the video material. Otherwise, missing values will be imputed as described below.

Physicians or decision coaches might not record their encounter with patients so that no video material for analysis will be available. If no part of the decision-making process has been recorded, the overall mean score values of MAPPIN-O_dyad_ will be imputed in the calculation of the primary endpoint. It is expected that only very few cases of imputation will occur (maximum of about 5 % in each group). A secondary sensitivity analyses will be performed after excluding patients with imputed values of the primary outcome.

Secondary outcomes will be analyzed with a complete case analysis. Questionnaires exceeding 20 % of missing values will be excluded from analysis [85]. Based on former studies, missing values should not be expected [78].

### Monitoring

For the individual woman, the study period will be 2 months. We do not expect adverse events. Hence, no safety board will be established, no interim analyses will be conducted, no stopping rules are defined, and no audits have been planned in advance.

### Strategies to improve study adherence and intervention fidelity

After study centers have included two patients, study centers (CG and IG) will be visited by one member of the research team to ensure that study procedures (form completion and video recordings of the encounters) are conducted as intended.

### Research ethics approval

Ethical clearance for the study has been obtained from the ethical committee of the German Society of Nursing Science (Deutsche Gesellschaft für Pflegewissenschaft DGP) (Request no. EK-15-003).

### Process evaluation

We will carry out a process evaluation to identify factors that might facilitate or interfere with the successful implementation of the intervention [52, 86]. The process evaluation contributes to a deeper understanding of the relation between the components of the intervention and the intervention effectiveness or failure. We used the guidance for process evaluation of cluster-randomized trials by Grant et al. [87]. It includes the following process evaluation components: trial delivery, intervention implementation and the responses of targeted participants. Following Grant et al. [87], we distinguish between clusters and individuals to address the specifics of a cluster-randomized trial. Furthermore, we will consider the domains: context of the study, applied theory and unintended consequences (see Additional file 2).

The objectives of our process evaluation are as follows:

1. describe our recruitment strategy and to identify reasons for participation and non-participation (clusters and individuals), so that a conclusion about the generalizability of the study results and implementation conditions can be made.
2. ensure that the training intervention (SPUPEO-workshop and training, SPUPEO is a German acronym “Spezialisierte Pflegefachkräfte zur Unterstützung einer informierten partizipativen Entscheidungsfindung in der Onkologie,” which means “qualified nurses to support SDM in oncology”) was delivered as intended because variations in intervention delivery might cause outcome differences between the clusters.
3. gather the attitude of health care professionals about decision coaching as an indicator for the response of clusters.
4. ensure that the decision coaching was delivered as intended (intervention fidelity) since variations in intervention delivery might cause outcome differences between the patients.
5. gather the attitude of patients about decision coaching and patient participation since it is associated with the acceptance of the intervention and the outcomes.
6. identify which intervention components work, which do not and why.
7. look for unintended consequences of the intervention.
8. identify differences between the clusters that might influence the effectiveness of our intervention.
9. identify structural barriers and facilitators of a successful implementation of decision coaching into the breast care center.

We chose an explanatory mixed-methods design to get a better conception of (inter-) dependencies and related factors that influence function and outcome of the complex intervention [88, 89].

After study completion, we will conduct telephone interviews with involved physicians and nurses. The interview will focus on potential barriers and facilitators that are revealed from the study results and previous results of the process evaluation.

## Discussion

The study aims at the evaluation of a structure changing complex intervention to improve informed SDM in breast care centers. As yet, decision coaches have not been implemented in standard care in oncology in the German healthcare system. An environmental scan of Légaré et al. [90] reveals that inter-professional approaches of shared decision-making are lacking. The intervention has advantages such as the low-threshold offer for patients and continuity of care. The strength of the study is that our outcome focuses on the mutual SDM behavior of all involved parties. However, we could not take into account the perceived SDM behavior of the involved parties in our primary outcome, since no validated compound measure for inter-professional SDM exists.

Our primary endpoint assesses the extent of SDM of the whole decision-making processes. Each process is based on two talks in the IG and one talk in the CG due to our structural intervention. Talking with two health care professionals may result in higher overall SDM performance. Hence, systematic differences in this outcome are possible. In the IG, the decision-making process is split. In this respect, focusing only one talk would skip part of the whole process. Applying one holistic measurement for the process in the IG will meet this doubt.

In addition, using MAPPIN’SDM for decision-making processes comprising two talks has not been validated yet. To estimate construct validity, interrater reliabilities will be computed and the consensus ratings of two independent raters will be used to calculate the primary endpoint.

The study has limitations, since blinding of clusters, women and assessors is not possible. Since the allocation of women is not concealed, a selection or participation bias may occur.

The potential selection bias of participating women is challenging. Physicians might not ask every eligible woman, because of implicit assumptions of eligibility for example, age or educational background. This issue will be discussed with all participating physicians. The number of eligible women will be monitored in all centers. In addition, strategies to speak to eligible women will be discussed and a guideline for physicians will be provided.

One further limitation is that some breast care centers already involve specialized nurses in counselling of women prior to the treatment decision. Because we only focus on the physician consultation in the control group, we might overlook the SDM behavior of nurses that would lead to an improvement of the extent of SDM in the control group. We will record if nurses are part of the usual counselling process prior to randomization, but the extent and quality of possible nurse counselling regarding SDM will not be quantified. Within the scope of process evaluation, we video tape these nurse encounters, too.

The video recordings might influence healthcare professionals due to the artificial situation. However, this effect might occur in both groups.

The cluster design has been chosen to avoid contamination between study arms. However, breast care nurses are organized in networks and might exchange information about the study intervention.

We defined seeking a second opinion as exclusion criterion. Women might have already been included in the study in the center they visited first and should not be asked for participation a second time.

In summary, the results will indicate if decision coaches will improve informed shared decision-making in oncology and which resources and conditions are necessary to implement the new model in certified breast care centers in Germany.

### Protocol amendments

Modifications or amendments that have an impact on the conduct of the study will be documented and unveiled in further publications.

### Confidentiality

All members of the research team will practice professional secrecy. We will comply with the federal states’ data protection laws. To ensure data privacy, pseudonyms are used that allow combining data sets and deleting of data if patients withdraw informed consent. The pseudonym list and informed consent forms are kept under lock at the participating study centers. Computer files are code-locked to prohibit unauthorized access. Anonymization of the video-material is not possible. Data that give hints to identification of individuals will not be assessed. Videos will be deleted after study completion. Anonymity of participants will be observed strictly within the publications.

### Access to data

The study center will coordinate the intra-study data sharing process. The cleaned data sets will be available for all principal investigators. In order to meet the requirements of the data sharing policy for clinical trials of the Institute of Medicine [91, 92], full access to raw data will be available on request.

### Dissemination policy

Results will be published in scientific journals. According to the recommendations of the International Committee of Medical Journal Editors (ICMJE), only persons directly involved in the study will be designated as authors [93].

## Trial status

Currently, the recruitment of breast care centers is ongoing. Patients will be recruited from July 2015 until March 2016.

## Additional files

Additional file 1:
**Completed SPIRIT checklist.** (DOCX 61 kb) Additional file 2:
**Domains of process evaluation and objectives.** (DOCX 19 kb)

## Abbreviations

BCN: breast care nurse
CG: control group
DCIS: ductal carcinoma in situ
DCS: Decisional Conflict Scale
DFG: German Research Foundation
EBPI: Evidence-based Patient Information
ICCC: intra-cluster-correlation coefficient
IG: intervention group
ITT: intention-to-treat
MAPPIN-SDM: Multifocal approach to the ‘sharing’ in shared decision-making
MAPPIN-O: Multifocal approach to the ‘sharing’ in shared decision-making, observer instrument
MAPPIN-Q: Multifocal approach to the ‘sharing’ in shared decision-making, questionnaire
MRC: Medical Research Council
PtDA: patient decision aid
SDM: shared decision-making
SPIRIT: Standard Protocol Items, Recommendations for Interventional Trials
SPUPEO: German acronym that means qualified nurses to support informed SDM in oncology
