# Supplementary material for: Informed shared decision-making supported by decision coaches for women with ductal carcinoma in situ: study protocol for a cluster randomized controlled trial
Source: Trials. 2015 Oct 12;16:452. doi: 10.1186/s13063-015-0991-8 (PMC4603943; doi:10.1186/s13063-015-0991-8)
Supplement: Additional file 2: — Domains of process evaluation and objectives. (DOCX 19 kb) [file 13063_2015_991_MOESM2_ESM.docx]

|  | **Objectives** | **Methods** | **Time point** |
| --- | --- | --- | --- |
| **Context** | Description of context factors | **Breast care centre**  *Questionnaire:*  Certification body (Oncomap, Eusoma)  Frequency of tumour boards  Number of specialized nurses (breast care nurses and oncology nurses)  Number of the treating physicians  Primary cases of DCIS in 2014  Primary cases of DCIS treated with breast conserving surgery in 2014  Primary cases of DCIS treated with mastectomy in 2014  Recommended radiotherapy after breast conserving surgery in 2014 (Target according to guideline: ≥95%)  *Interview / protocol / visitation*  Description of the organizational process and structures: e.g. how are the nurses integrated into patient´s care, are there fixed surgery days, how are physician encounters arranged?  Interconnectedness with other clusters or subgroups e.g. nurses? What patient information material is given to patients with DCIS routinely?  **Nurses**  *Questionnaire:*  Working experience of the specialized nurses (further education and trainings)  Working time  Release of clinical activities on the ward (in % of the total working time) and absolute in hours  Self-efficacy  Do nurses deliberate patients in routine care?  Possible contamination: Is the nurse member of a (regional) BCN-network?  Risk knowledge on DCIS (Baseline)  **Physicians**  *Questionnaire*  Working experience  Qualification in senology | -t_4_, -t_3_ |
| **Recruitment of clusters** | Description of recruitment | *Recruitment sheet:*  Assessment of the reasons for participation or nonparticipation  Documentation of recruitment process (description) | -t_6_ |
| **Delivery to clusters** | Development and pretest of the intervention components (SPUPEO-training program for nurses, SPUPEO-Workshop for physicians, ptDA) | *Questionnaires, observation and short interviews:*  Comprehensibility, plausibility and acceptance of the materials | Before the main trial |
|  | Feasibility and pilot study in 2 Breast care centres in Berlin | *Questionnaires, observation and short interviews:*  Comprehensibility, plausibility and acceptance of the materials | Before the main trial |
|  | Intervention fidelity   - Precise training schedule (see curriculum) - Presence of the participating nurses - Presence of the participating physicians - Frequency and intensity of accompanying supervision and feedbacks / study nurse contacts - Evaluation of the training program | **SPUPEO-training programme:**  *Documentation* of the training programme, *Attendance list*  *Questionnaire:* Nurses´ knowledge and attitude about the workshop  **Physician Workshop**  *Documentation* of the workshop  *Questionnaire:* Attitude of the physicians about the workshop | -t_2_ |
|  | Provision of information about the study process | Protocol of the kick off-meeting | -t_4_ |
| **Response of clusters** | Attitude about decision coaching | *Questionnaire:* Nurses´ attitude  *Questionnaire:* Attitude of the physicians  *Interviews* with physicians and nurses and main stakeholder  *Interviews* with SDM-thwarting physicians to explore their motivation | -t_2_  t_7_ |
| **Recruitment and reach of individuals** | Description of recruitment | *Recruitment sheet:*  Assessment of the reasons for participation or nonparticipation  Assessment of primary DCIS cases in the study period (comparison with amount of included patients) | -t_1_ |
| **Delivery to individuals** | Fidelity (Use of materials as intended within the decision coaching encounters and information fidelity) | *Videotapes* (Fidelity) + first 2 Coaching encounters under supervision (*documentation* of the feedbacks)  *Documentatio*n   - Duration of the coaching encounter sessions - Number of coaching encounter sessions - Distance between coaching encounter sessions   *Copy of the patient decision guidance* (used as intended by nurses and physicians)  Recommendation of the tumour board  Continuity of the health care professionals during the coaching process | t_3_ |
| **Response of individuals** | Patients´ attitude about decision coaching  Use of the offered support interventions (psycho-oncological support, second opinion) | *Questionnaires*  Patients’ attitude about the decision coaching  *Documentation*  Use of psycho-oncological support in the breast care centre  Obtaining a second opinion | t_2_, t_4_  t_6_ |
| **Maintenance** |  | Video recordings that will be analysed  *Copy of the patient decision guidance* | t_3_,t_4_ |
| **Unintended consequences** |  | Exchange with other clusters (networks e.g.)  Change of organizational structures  Barriers and facilitators of the implementation of decision coaches in the breast care centres | continuously |
| **Theory** |  | Theory of Planned Behaviour |  |

Domains of process evaluation and objectives
